# Robo4 inhibits gamma radiation-induced permeability of a murine microvascular endothelial cell by regulating the junctions

**DOI:** 10.1186/s11658-022-00413-w

**Published:** 2023-01-16

**Authors:** Seyram Yao Adzraku, Guozhang Wang, Can Cao, Yurong Bao, Yizhou Wang, Alhaji Osman Smith, Yuwei Du, Haiyang Wang, Yue Li, Kailin Xu, Jianlin Qiao, Wen Ju, Lingyu Zeng

**Affiliations:** 1 Blood Diseases Institute, Xuzhou Medical University, Xuzhou, 221002 China; 2 Key Laboratory of Bone Marrow Stem Cell, Xuzhou, 221002 Jiangsu China; 3 Department of Hematology, The Affiliated Hospital of Xuzhou Medical University, Xuzhou, 221002 China; 4 Xuzhou Ruihu Health Management Consulting Co., Ltd, Xuzhou, 221002 China

**Keywords:** Robo4, Microvascular endothelial cells, Endothelial junctions, Irradiation, Permeability

## Abstract

**Background:**

Hematopoietic stem cell transplantation involves irradiation preconditioning which causes bone marrow endothelial cell dysfunction. While much emphasis is on the reconstitution of hematopoietic stem cells in the bone marrow microenvironment, endothelial cell preservation is indispensable to overcome the preconditioning damages. This study aims to ascertain the role of Roundabout 4 (Robo4) in regulating irradiation-induced damage to the endothelium.

**Methods:**

Microvascular endothelial cells were treated with γ-radiation to establish an endothelial cell injury model. Robo4 expression in the endothelial cells was manipulated employing lentiviral-mediated RNAi and gene overexpression technology before irradiation treatment. The permeability of endothelial cells was measured using qPCR, immunocytochemistry, and immunoblotting to analyze the effect on the expression and distribution of junctional molecules, adherens junctions, tight junctions, and gap junctions. Using Transwell endothelial monolayer staining, FITC-Dextran permeability, and gap junction-mediated intercellular communication (GJIC) assays, we determined the changes in endothelial functions after Robo4 gene manipulation and irradiation. Moreover, we measured the proportion of CD31 expression in endothelial cells by flow cytometry. We analyzed variations between two or multiple groups using Student’s *t*-tests and ANOVA.

**Results:**

Ionizing radiation upregulates Robo4 expression but disrupts endothelial junctional molecules. Robo4 deletion causes further degradation of endothelial junctions hence increasing the permeability of the endothelial cell monolayer. Robo4 knockdown in microvascular endothelial cells increases the degradation and delocalization of ZO-1, PECAM-1, occludin, and claudin-5 molecules after irradiation. Conversely, connexin 43 expression increases after silencing Robo4 in endothelial cells to induce permeability but are readily destroyed when exposed to 10 Gy of gamma radiation. Also, Robo4 knockdown enhances Y731-VE-cadherin phosphorylation leading to the depletion and destabilization of VE-cadherin at the endothelial junctions following irradiation. However, Robo4 overexpression mitigates irradiation-induced degradation of tight junctional proteins and stabilizes claudin-5 and ZO-1 distribution. Finally, the enhanced expression of Robo4 ameliorates the irradiation-induced depletion of VE-cadherin and connexin 43, improves the integrity of microvascular endothelial cell junctions, and decreases permeability.

**Conclusion:**

This study reveals that Robo4 maintains microvascular integrity after radiation preconditioning treatment by regulating endothelial permeability and protecting endothelial functions. Our results also provided a potential mechanism to repair the bone marrow vascular niche after irradiation by modulating Robo4 expression.

## Introduction

Total body irradiation (TBI) is commonly used to treat blood malignancies before hematopoietic stem cell transplantation (HSCT) [1, 2]. Even though the vasculature is not the primary target, it is adversely affected by the irradiation treatment. This preconditioning treatment is associated with several acute and chronic debilitating effects, including poor graft function (PGF) due to electrolyte imbalance and long-term hypoglycemia [3]. While much emphasis is on the reconstitution of an adequate number of HSCs to repopulate the hematopoietic system after HSCT, endothelial cell (EC) preservation and normal functioning are indispensable to overcoming the radiation preconditioning damage.

Under steady conditions, the mature vascular endothelium is stable and quiescent, and ECs are held together through junctional proteins [4, 5]. These junctions maintain firm contact between cells, but under certain pathological or stressful conditions, the integrity of the vascular endothelium is impaired, affecting its function [6, 7]. In our previous study, we found that the pretreatment in HSCT with high-dose irradiation and chemotherapy could remove many tumor cells and empty the bone marrow cavity while also causing the integrity of the bone marrow sinusoidal niche to be destroyed [8]. However, exposure to ionizing radiation (IR) causes inflammation of the endothelium and vascular fibrosis, contributing to endothelial dysfunction [9]. IR causes increased endothelial monolayer permeability, but the mechanisms that mediate this phenomenon are poorly understood [10, 11]. It is, however, imperative that radiation exposure might affect endothelial integrity by acting on junctions between ECs directly or indirectly [12, 13]. The barrier permeability is regulated by endothelial cell–cell adhesions, including tight junctions (TJs), adherens junctions (AJs), and gap junctions (GJs) [4, 5, 14]. TJ adhesion is mediated mainly by occludin and claudins linked to zonula occludens (ZO) and other protein complexes, which mediate the interaction between the adhesion molecules and actin filaments [7, 15]. VE-cadherin is a crucial transmembrane component of endothelial AJs and is expressed mainly in ECs necessary for endothelial barriers’ complete formation and function. VE-cadherin is connected indirectly via its cytoplasmic tail to actin filaments by a complex of proteins, including α- and β-catenins, plakoglobin (γ-catenin), and p120-catenin, which are essential for junctional stability and also needed for the dynamic opening and closure of junctions [16–18]. Factors that induce barrier permeability activate the phosphorylation of VE-cadherin, leading to its internalization and subsequent degradation [19–21]. GJs, formed by the interaction of two connexons from opposite cells, provide a means to facilitate direct cell-to-cell transfer of signaling molecules, ions, current, and transmembrane potential in normal and permeability-induced ECs. Ideally, six connexins oligomerize to form a hemichannel (connexon). Three main connexin (Cx) isoforms are expressed in ECs: Cx37, Cx40, and Cx43. These GJs are responsible for the interaction between endothelial and endothelial-smooth muscle cells [22–24]. The effects of IR on connexins remain to be elucidated.

Robo4 is an endothelial-specific receptor involved in EC migration, proliferation, angiogenesis, and the maintenance of vasculature integrity [25]. Robo4 interacts with the transmembrane receptor Unc5B activating its downstream signaling to suppress VEGF receptor 2-phosphorylation and vascular leakage. Slit2 also binds Robo4 to inhibit VEGF-induced EC migration by regulating functions of Robo1 and GTPases such as Rac1 and Cdc42. On the other hand, the C-terminal compartment of Robo4 modulates EC functions by interacting with the focal adhesion-associated protein, also called paxillin, and the cytoskeleton-regulating proteins and Wiskott-Aldrich syndrome protein [25, 26]. Recently, Robo4 was established as a modulator of endothelial permeability and integrity in response to lipopolysaccharide (LPS), VEGF, or TNF-α-induced permeability [27–30]. Under osmotic stress, Robo4 expression in the retinal microvascular EC is upregulated by hyperglycemia contributing to endothelial dysfunction [31]. Hyperglycemia-induced Robo4 overexpression in ECs is mediated by the transcription factor, specificity protein 1 (SP1), bearing two binding sites in the promoter region of Robo4 that improve transcriptional levels of Robo4. This SP1/Robo4 signaling pathway regulates the endothelial monolayer migratory ability, permeability, and angiogenesis in vitro [32].

On the contrary, Robo4 is downregulated in retinal microvascular EC under hypoxic conditions suggesting that Robo4 may exhibit different functions in various cell types following stress-induced injury [33]. Some studies identified hypoxia-inducible factor-1 (HIF-1) as the transcription factor that modulates the expression level of Robo4 in ECs under hypoxia [29, 33]. However, the role of Robo4 in regulating the irradiation-induced permeability of ECs is unknown. This study will investigate the importance of Robo4 in maintaining the integrity of microvascular ECs after radiation treatments.

## Materials and methods

### Cell culture

bEnd.3 cells (ATCC^®^ CRL-2299™) were cultured in Dulbecco’s modified Eagle medium (DMEM, Gibco, catalog number: C11995500BT) supplemented with 10% fetal bovine serum (FBS, Gibco, catalog number: 10099-141) and 1% penicillin/streptomycin (Gibco; Thermo Fisher Scientific, Inc., Waltham, MA, USA) in a humidified 5% CO_2_ incubator at 37 °C and used for at most ten passages.

### Irradiation

Microvascular ECs were seeded at 1 × 10^4^ cells/cm^2^ density and treated with gamma (γ) radiation after reaching 80% confluence using a GSR C1 137 cesium gamma irradiator (Gamma-Service Medical, Bautzner, Germany) at a dose of 10 Gy with a dose rate of 1.88 Gy/min. The irradiated EC cells were cultured in humidified 5% CO_2_ incubators at 37 °C until further analysis. Non-irradiated control samples were treated similarly (i.e., culture medium, transport to the accelerator, and incubation conditions).

### Preparation of plasmids

Mouse Robo4 was amplified by PCR using the specific primers (Invitrogen, Carlsbad, CA, USA) containing Age 1 (CCGG) and EcoR 1 (AATTCAAAAA) restriction sites. The synthesized mRobo4 oligonucleotides: 5′-GCTGACTGTGTCTTCACTGAT CTCGAG ATCAGTGAAGACACAGTCAGC-3′ (shRobo4#1), 5′-GCCAACAACCTATGGCTATAT CTCGAG ATATAGCCATAGGTTGTTGGC-3′ (shRobo4#2) and 5′-GCCACCAACAATGCTGGGCAA CTCGAG TTGCCCAGCATTGTTGGTGGC-3′ (shRobo4#3) with or without the 3′-UTR (TTTTTG) were inserted and cloned into GV248 vector (hU6-MCS-Ubiquitin-EGFP-IRES-puromycin), (Genechem, Shanghai, China) following Genechem standard procedures and confirmed by restriction digestion and DNA sequencing. A plasmid carrying a non-targeting control sequence (TTCTCCGAACGTGT CACGT) was used as a transfection control (shCont.). For overexpression, lentiviral vectors harboring Robo4 overexpression vectors (Robo4-OX) and its control vector (CON) were obtained from Genechem. The recombinant GV 248 vector plasmid (20 μg) containing shRobo4 and Robo4-OX vectors, 15 μg pHelper 1.0 vector plasmid (pGAG-POL), and 10 μg pHelper 2.0 vector plasmid (pCMV-VSVG) were co-transfected into the packaging cell line 293 T cells under the mediation of Lipofectamine. Viral supernatant collected from the HEK293T cells two days after transfection were passed through a 0.45 μm syringe filter (Thermo Fisher) and ultracentrifuged (Beckman).

### Stable establishment of Robo4 knockdown and overexpression in microvascular ECs

Microvascular ECs (3 × 10^5^) were plated to attain 60–70% confluence in a 60 mm culture dish and allowed to adhere overnight. The shRobo4, Robo4-OX, and corresponding control vectors were transduced with 1× HitransG P (Genechem, Shanghai, China) in 10% FBS DMEM into the adherent ECs. After 72 h, infected lentiviral cells were selected with 1 ug/ml puromycin (Genechem, Shanghai, China), and GFP positivity and transduction levels were checked using fluorescent microscopic and FACS analysis. Target expression alteration was assessed by quantitative real-time PCR (qRT-PCR) and western blot.

### Total RNA isolation and quantitative reverse transcription-polymerase chain reaction (qRT-PCR)

Total RNA was isolated from the adherent microvascular EC monolayer 24 h post-irradiation using TRIzol reagent (Invitrogen/Life Technologies, Carlsbad, CA, USA). After quantification using a NanoDrop 2000c spectrophotometer (NanoDrop Technologies, Rockland, DE, USA) to obtain an OD ratio of 260/280 between 1.8 and 2.0, reverse transcription was performed with 1.0 µg RNA using PrimeScript reverse transcriptase master mix (Takara Biotechnology, Dalian, China). During qRT-PCR, samples were prepared in triplicates in 96-well plates. Each well contained 2.0 μL of cDNA, 200 nM forward primer, 200 nM reverse primer, 1 × SYBR Green I Master mix, and up to 20 μL of DNase/RNase-free distilled water. The 96-well plates were loaded into a Roche LightCycler 480 II real-time PCR System (Roche Life Sciences). The following PCR cycling program was used: 10 min at 95 °C, 40-cycles of 30 s at 95 °C, 30 s at 60 °C and 15 s at 72 °C; 15 s at 95 °C using LightCycler^®^ 480 software 1.5.0 SP4 for the analysis. The primers, designed with NCBI Primer-BLAST and Primer3 prepared by Thermo Fisher Scientific and Invitrogen, were used in this study as tabulated in Table 1. Triplicate real-time PCR analyses were performed for each sample, and the resulting threshold cycle (CT) values were averaged. Target mRNA expression was normalized to the averaged expression of the housekeeping genes (GAPDH and beta-actin), yielding the ∆CT value. Each target gene’s relative mRNA expression levels were calculated by 2^−∆∆Ct^.

**Table 1:** Real-time quantitative PCR primer sequences

Genes	Primer sequences	
	Forward 5’ → 3’	Reverse 5’ → 3’
Robo4	TTATGGCTCCCTCATCGCTG	GAGGCTGTCTGAGCTGGAAC
ZO-1	CAGCCGGTCACGATCTCCT	TCCGGAGACTGCCATTGC
Occludin	CACACAGGACGTGCCTTCAC	GAGTATGCCATGGGACTGTCAA
Claudin 5	CTGCTGGTTCGCCAACATT	TGCGACACGGGCACAG
PECAM-1	CCAAAGCCAGTAGCATCATGGTC	GGATGGTGAAGTTGGCTACAGG
Connexin 43	TACCACGCCACCACTGGC	AATCTCCAGGTCATCAGG
Connexin 40	TTTGGCAAGTCACGGCAGGG	TTGTCACTGTGGTAGCCCTGAGG
Connexin 37	GGCTGGACCATGGAGCCGGT	TTTCGGCCACCCTGGGGAGC
VE-cadherin	GAAGCCTCTGATTGGCACAGTG	TTTTGTGACTCGGAAGAACTGGC
γ-Catenin	ACCAGCATCCTGCACAACCTCT	GGTGATGGCATAGAACAGGACC
β-Catenin	CACAAGCAGAGTGCTGAAGGTG	GATTCCTGAGAGTCCAAAGACAG
β-Actin	ATGTGGATCAGCAAGCAGGA	AAGGGTGTAAAACGCAGCTCA
GAPDH	CATGGCCTTCCGTGTTCCTA	GCGGCACGTCAGATCCA

### Antibodies

Primary antibodies against ZO-1 (Cat. No. 21773-I-AP), CD31 (Cat. No. 28083-I-AP), ROBO4 (Cat. No. 20221-I-AP), connexin 43 (Cat. No. 26980-I-AP), and beta-actin (Cat. No. 20536-I-AP) were purchased from Proteintech Group, Inc (Rosemont, IL, USA). Rabbit polyclonal claudin 5 (Cat. No. AF5216) and P-VE-cadherin (Cat. No. AF3265) antibodies were purchased from Affinity Biosciences (Cincinnati, OH, USA). In addition, rabbit polyclonal anti-occludin (Cat. GB11149-2) and rabbit polyclonal anti-β-tubulin (Cat. No. 2128S) were purchased from Servicebio (Nanjing, China) and Cell Signaling Technology (Danvers, MA, USA), respectively. Alexa Fluor^®^ 594 anti-rabbit secondary antibody (Cat. no. ab15008), goat pAb to IgG (FITC) (Cat. no. ab97050), and rabbit monoclonal anti-VE-cadherin (Cat. No. ab205336) were also purchased from Abcam (Cambridge, MA, USA). The anti-rabbit IgG HRP-linked antibody (Cat. 7074) was purchased from Cell Signaling Technology (Danvers, MA, USA).

### Protein isolation and immunoblot analysis

To extract proteins, 200 μL of ice-cold RIPA lysis buffer (Beyotime, China), comprising 150 mM NaCl, 50 mM Tris–HCl pH 7.4, 1% NP-40/IGEPAL CA-630, 0.5% sodium deoxycholate, 0.1% SDS, phosphatase and protease (PMSF) inhibitors (Solarbio^®^, China), was added to the adherent monolayer ECs (10^6^ cells) 24 h post-radiation and incubated for 5 min on ice. Next, adherent cells were gently scraped and transferred into sterile 1.5 mL EP tubes. The cell lysates were collected at 15,000 rcf for 20 min at 4 °C, and the protein concentrations were evaluated using a bicinchoninic acid (BCA) Protein Assay Kit (Beyotime, China). Subsequently, the protein lysates were supplemented with Laemmli buffer (one fourth of the total volume) (Beyotime, China) and heated at 95 °C for 5 min. 20 μg of the protein lysates were separated using 6–12% SDS-PAGE and blotted onto polyvinylidene difluoride (PVDF) membranes (Life Science, Germany) in a tank blot unit (Mini-PROTEAN II, Bio-Rad, Hercules, CA, USA). The blotted membranes were blocked for 1 h at room temperature using either 5% non-fat dry milk (BD, Le Pont de Claix, France) or 5% BSA (Solarbio^®^, China) dissolved in a tris-buffered saline solution containing 0.1% (v/v) Tween 20 (Sigma-Aldrich Co. LLC, St. Louis, MO, USA). Afterward, membranes were incubated overnight at 4 °C with the appropriate primary antibody. After washing with 1× TBST, the membranes were incubated for 1 h at room temperature with the appropriate horseradish peroxidase (HRP)-conjugated secondary antibodies (CST, Danvers, MA, USA). The HRP-immunoreactive bands were detected with the enhanced chemiluminescence (ECL) detection kit (GE Healthcare Life Sciences, Little Chalfont, UK). Band signal intensities were analyzed using the Fiji software. Protein loading was normalized by β-actin or β-tubulin protein expression.

### Immunofluorescence staining

Microvascular ECs (20,000 cells) with or without Robo4 gene manipulation were seeded on 12-mm round coverslips in 24-well plates and incubated for 24 h to attain 60–70% confluence in a humidified 5% CO_2_ incubator at 37 °C. Twenty-four hours after irradiation treatment, the cells were washed with phosphate-buffered saline (PBS), fixed with 4% paraformaldehyde or 100% ice-cold methanol for 10 min at room temperature, permeabilized for 15 min with 0.1% (v/v) Triton X-100. After three washes with PBS, the cells were blocked with 1% BSA, 22.52 mg/mL glycine in PBST (PBS + 0.1% Tween 20) at room temperature for 30 min. Coverslips were incubated with primary antibodies for claudin-5, connexin-43, VE-cadherin, and ZO-1 overnight at 4 °C. After three additional washes with PBST (5 min/wash), cells were incubated with secondary antibodies conjugated with Alexa Fluor^®^ 488 or Alexa Fluor^®^ 594 (1:500 dilution) at room temperature for 2 h in a moist chamber while protected from light. Lastly, the cells were stained with 1 mg/ml of 4′,6-diamidino-2-phenylindole (DAPI) for nuclei labeling after adequate rinsing in PBS. The coverslips were mounted with an anti-fading mounting medium, and the fluorescent signals were detected using a Zeiss confocal fluorescence microscope (ZEISS LSM 880, Munich, Germany). We used Fiji software (Bethesda, MD, USA) to analyze the acquired images. Figures were assembled with Adobe Illustrator. Adjustments of brightness and contrast were performed in the figure preparations. For an accurate comparison, sample images of the same antigen were acquired under constant acquisition settings.

### Flow cytometry

Following plasmid transfection and γ-radiation exposure, microvascular ECs (5 × 10^5^ cells) inoculated in a 60-mm sterile dish were incubated at 37 °C, 5% CO_2_ for 24 h. The resulting cells were washed with PBS and harvested using a 0.25% trypsin/EDTA reagent. The cells were then incubated with R-phycoerythrin (PE)-conjugated rabbit anti-mouse CD31 (16B1; Thermo Fisher Scientific, Waltham, MA, USA) or PE-conjugated rabbit IgG1 (Thermo Fisher Scientific) for 30 min in the dark at 4 °C and then washed with ice-cold PBS containing 2% FBS. The fluorescence intensity of the cells was determined by a flow cytometer (BD LSRFortessa; BD Biosciences, San Jose, CA, USA), and the data obtained were analyzed by FlowJo version 10 software.

###  Cycloheximide chase assay

The turnover of VE-Cadherin protein was evaluated using cycloheximide inhibition of protein synthesis. Cells were treated with 25 µg ml^−1^ cycloheximide (Aladdin Biochemical Tech., Shanghai, China) and lysed at 0, 6, and 12 h time points. The expression of VE-cadherin was analyzed by immunoblotting.

### Cell counting kit-8 (CCK-8)

Following Robo4 gene manipulation, microvascular ECs (1 × 104/well) were plated in 96-well plates and subjected to 10 Gy gamma radiation. Twenty-four hours post-irradiation, 10 μL cell counting kit-8 (Beyotime, Shanghai, China) was added, and cells in the culture plate were incubated for 4 h. The absorbance was measured at 450 nm with a microplate reader (BioTek, Synergy H1 microplate reader; Winooski, VT, USA).

### Endothelial permeability assessment

The endothelial permeability assay was performed in 24-well plates containing 12 Transwell inserts (6.5-mm diameter, 0.4-mm pore size polycarbonate filters, Corning Costar Corporation), according to the manufacturer’s instructions. One hundred thousand microvascular ECs with or without Robo4 gene manipulation were seeded and cultured in a complete growth medium for 72 h before being exposed to gamma radiation. Twenty-four hours post-irradiation, the medium was removed entirely from apical and basal compartments, 500 µL of fresh medium was added to the bottom chamber, and 150 µL of medium containing 10.0 µg/mL 40 kDa FITC-dextran (MW4000, Sigma, Shanghai, China) was loaded into the apical compartment of inserts. Aliquots of 100 μL were removed from the basolateral chamber every 30 min for 2 h after the application of FITC-dextran and transferred into a black 96-well plate to quantify the fluorescence intensity (excitation and emission at 485 and 535 nm, respectively) using a fluorescence spectrophotometer (BioTek, Synergy H1 microplate reader, Winooski, VT, USA). The permeability assay of the endothelial monolayer was repeated three times for each time point for three samples. The following equation calculated the absolute permeability *P* [cm/s]: *P* = [*C*(*t*) – *C*(*t*_0_)] × *V*/*A* × *t* × *C*_0,_ where *C*(*t*) is the concentration [μg/ml] of FITC-dextran in the samples that were taken from the basal chambers after 30, 60, 90 or 120 min. *C*(*t*_0_) is the FITC-dextran concentration [μg/ml] of the samples at 0 min, and *t* is the duration of the flux(es). *V* is the volume [cm^3^] in the lower compartment; *A* is the surface of the Transwell^®^ membrane [cm^2^]. *C*_0_ is the initial concentration [μg/ml] of FITC-dextran in the apical chamber.

### Endothelial monolayer staining

 EC suspensions of 200 μL were added to Transwell inserts at a density of 5 × 10^5^ cells/mL and incubated for 72 h to form monolayer confluence before irradiation. Twenty-four hours later, media from the inserts were carefully removed without disturbing the cell monolayer. Afterward, 100 μL of 0.5% crystal violet staining solution was added to the insert, covered, and kept at room temperature for approximately 20 min. After removing the cell stain, the inserts were rinsed twice with PBS, filling the insert and receiver plate well with 200 μL and 1 ml of washing buffer. Stained cells were brightfield-imaged at 5× objective magnification on a Nikon Eclipse Ti inverted microscope, and data were analyzed with NIS-Elements AR Microscope Imaging Software (Nikon, Japan).

### Gap junction-mediated intercellular communication (GJIC) assay

Semi-confluent microvascular ECs were treated with 10 Gy of γ-radiation. After 24 h, the irradiated cells were co-cultured with double stained (2 µM PKH26/3 µM Calcein Blue AM; MK Biotechnology, Shanghai, China) donor ECs in the ratio of 1:50 donor/recipient. After a co-culture duration of 4 h, cells were detached by Accutase, and the single-cell suspension was evaluated by BD LSRFortessa (BD Biosciences, San Jose, CA, USA) and analyzed using FlowJo v10 software. Calcein (blue) positive and PKH26 (orange) negative cells represent the cell population that has established GJIC.

### Statistical analysis

Results analysis was performed using GraphPad Prism^®^ 8.0 to conduct statistical analysis and draw graphs. The analyzed results were presented as the mean ± standard deviation (*x* ± std.). The variations between the two groups were analyzed using the non-parametric Student *t*-test for variable data and analysis of variance (ANOVA) for comparison between multiple data sets. Confidence level at *α* = 0.05 (degree of freedom), *P* < 0.05 indicates a statistically significant difference.

## Results

### Ionizing radiation disrupts junctions in microvascular ECs

**Fig. 1:**
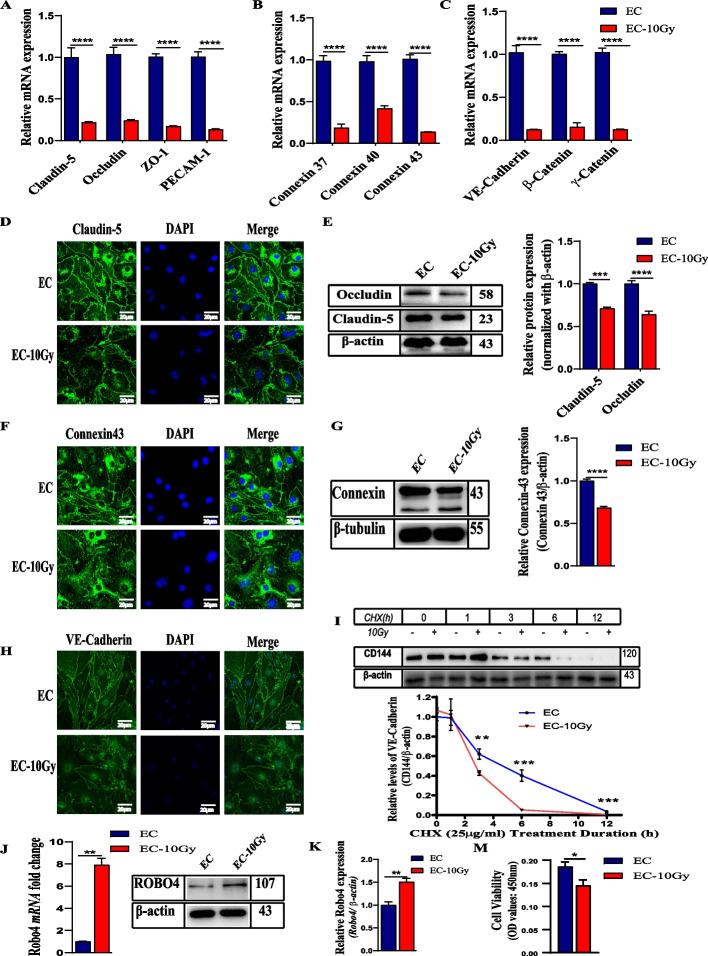
IR decreases the expression of EC junctional molecules but upregulates Robo4 expression. **A–C** RT-qPCR shows the relative mRNA expression of the TJs, GJs, and AJs. Gene expression results were normalized to glyceraldehyde-3-phosphate dehydrogenase (GAPDH) and beta-actin endogenous control and represented as fold change in gene expression. The error bars stand for the standard deviation from three separate independent experiments. **D** and **E** Detection of TJs (claudin-5 and occludin) protein expression by immunocytochemistry and western blot, respectively. **F** and **G** Analysis of gap junction (connexin 43) protein expression using immunofluorescence and immunoblotting. **H** and **I** The distribution and expression of VE-cadherin analysis with immunofluorescence, cycloheximide chase experiment, and western blot. **J** and **K** mRNA and protein detection of Robo4 in microvascular ECs after irradiation using RT-qPCR and WB. **M** Effect of irradiation on cell survival and proliferation of ECs. For all experiments, scale bars = 20 µm. *n* ≥ 3, and error bars represent std. *P < 0.05, **P < 0.01, ***P < 0.001, ****P < 0.0001

As cell junctions play an essential role in regulating vascular permeability [4, 5], we investigated whether IR treatment affects the integrity of microvascular EC junctions. Quantitative PCR analysis of crucial members of TJs (occludin, claudin-5, ZO-1, and PECAM-1), AJs (VE-cadherin, β-catenin, and γ-catenin), and GJs (connexin 37, 40 and 43) showed a significant reduction in mRNA expression levels of the EC junctional molecules 24 h after 10 Gy γ-radiation exposure (Fig. 1A–C). Immunofluorescence analysis of claudin-5, connexin 43, and VE-cadherin revealed increased deterioration of cell junctions 24 h post-irradiation (Fig. 1D, F, H). Also, immunoblotting analyses of lysates prepared from microvascular EC monolayers showed that claudin-5 and connexin 43 protein levels substantially decreased due to irradiation treatment (Fig. 1E, G). Using cycloheximide chase assay, we detected VE-cadherin protein turnover after irradiation. The results indicate a considerable decrease in VE-cadherin expression after IR (Fig. 1I), suggesting that irradiation primarily affects the distribution and total expression of VE-cadherin.

### The expression of ROBO4 increases in irradiated microvascular ECs

Robo4 is an endothelial-specific receptor regulating vasculature homeostasis under pathological conditions [25]. Therefore, microvascular ECs treated with high dose gamma radiation of 10 Gy at a dose rate of 1.88 Gy/min were investigated for mRNA and protein expression of Robo4 by RT-qPCR and western blot analyses, respectively. As described in Fig. 1 J and K, levels of the Robo4 molecule significantly increase (*p* < 0.01) after IR exposure, suggesting that Robo4 may involve in the regulation of vascular injury after irradiation. While there was upregulation of Robo4, the overall cell survival 24 h post-irradiation decreased (Fig. 1M).

### Reduced expression of Robo4 aggravates IR-induced injury by the disruption of AJs in ECs

**Fig. 2:**
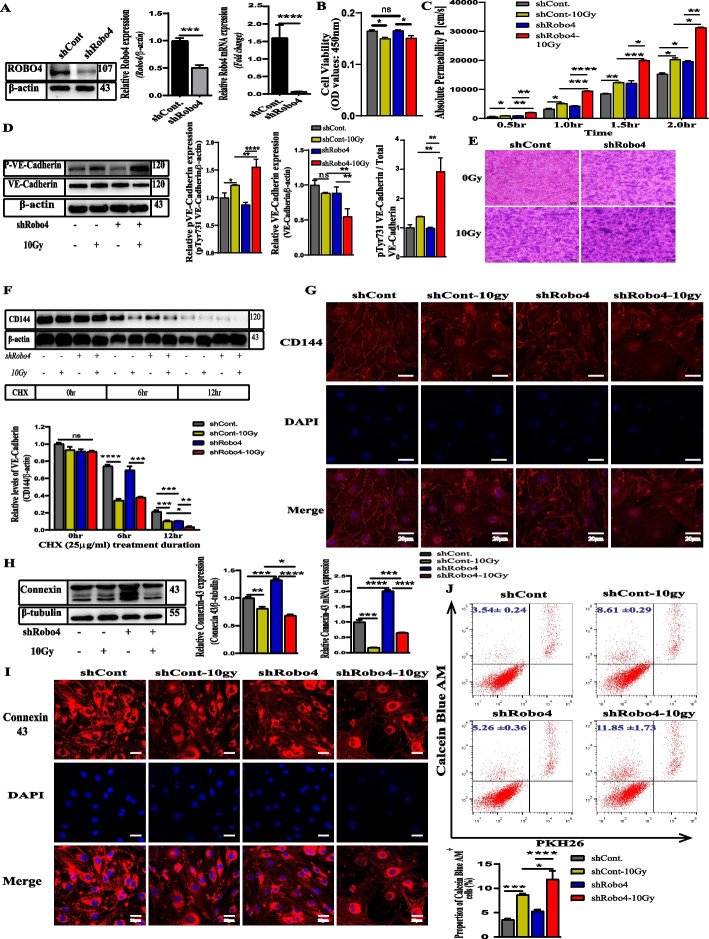
Robo4 knockdown depletes the VE-cadherin expression and increases EC monolayer permeability following irradiation. Establishment of a microvascular ECs line with Robo4 stable knockdown. **A** Western blot and RT-qPCR analysis to confirm the reduced expression of Robo4 using beta-actin as an endogenous control. **B** Effect of Robo4 silencing and irradiation on cell survival and proliferation of ECs. **C** and **E** Fitc-labeled dextran-40 kDa permeability assay and Transwell endothelial monolayer crystal violet staining results following Robo4 knockdown and gamma radiation treatment. **D** Immunoblotting analysis of total and phosphorylated Y-731 VE-cadherin in Robo4 silenced microvascular ECs after irradiation; The relative protein expression of phosphorylated Y-731 and total VE-cadherin normalized with endogenous beta-actin protein; the ratio of phosphorylated Y-731 VE-cadherin to total VE-cadherin using image J software for quantification. Cycloheximide treatment and immunoblotting were conducted as in (**F**). The CD144 band intensity was normalized to actin and then normalized to the *t* = 0 controls. **G** Confocal microscopic images of VE-cadherin (red) and 4’,6-diamidino-2-phenylindole (DAPI) staining (blue). **H** Western blot and qPCR analysis showing connexin 43 expression levels in Robo4 knockdown microvascular ECs following gamma radiation treatment. **I** Confocal images show immunofluorescence of connexin 43 (red) in Robo4 knockdown microvascular ECs, with or without irradiation. Nuclei appear in blue. Scale bars = 20 µm. **J** Flow cytometric analysis to determine endothelial GJs coupling ability in vitro. For all experiments, *n* ≥ 3, and error bars represent std. *P < 0.05, **P < 0.01, ***P < 0.001, ****P < 0.0001

To further establish the functional importance of Robo4 in regulating the vessel permeability in irradiation-injured ECs, we next knocked down the Robo4 gene in bEnd3 cells transduced with lentivirus expressing shRNA against Robo4. We confirmed these changes in its expression via western blotting and qPCR (Fig. 2A). Cell survival assessment of the microvascular ECs following Robo4 knockdown and irradiation shows that Robo4 downregulation does not significantly influence EC survival and proliferation (Fig. 2B). FITC-dextran permeability and endothelial monolayer staining assays to evaluate the impact of 10 Gy gamma radiation on Robo4 knockdown ECs and the control cells showed that IR induces permeability across the EC monolayer. However, the degree of permeability significantly increased in the Robo4 KD group following IR compared to the irradiated control group (Fig. 2C and E). In addition to degradation, irradiation leads to the delocalization of VE-cadherin in Robo4 knockdown EC monolayers. As mentioned above, in contrast to the similar expression of total VE-cadherin protein between radiated and non-irradiated control groups, the expression level of VE-cadherin in shRobo4 ECs reduced significantly after irradiation. Treating ECs with inflammatory stimuli could correlate with the phosphorylation of VE-cadherin tyrosine residues and a decrease in the stability of AJs [20]. Western blot analysis of the phosphorylation of VE-cadherin at the Y731 position shows an upregulation after irradiation in the shRobo4 ECs group (Fig. 2D). Cycloheximide chase assay was carried out following Robo4 knockdown and irradiation, cycloheximide was added three hours later, and the cells were incubated for 0, 6, and 12 h. As shown in Fig. 2F, the half-life of VE-cadherin protein was decreased by Robo4 depletion. From our immunocytochemical staining, Robo4 knockdown causes the downregulation of VE-cadherin expression after irradiation. It induces delocalization of VE-cadherin as described in (Fig. 2G). Robo4 KD exacerbates the degradation of AJs, especially CD144, after IR. These findings suggest that Robo4 may play a crucial role in regulating the vascular barrier permeability through the AJs after radiation injury.

### Silencing Robo4 in ECs stimulates GJs expression and promotes the damaging effects of IR

ECs express connexins that exert protective effects in an intrinsic and extrinsic manner [34]. To further establish the importance of Robo4 in microvascular ECs, we examined the mRNA and protein expression levels of connexin 43 following the knockdown of Robo4 using qPCR and immunoblotting analysis, respectively. Results indicated an increase in the mRNA expression level of connexin 43 after Robo4 knockdown. This finding was corroborated by the protein expression of connexin 43, suggesting that lentiviral-mediated silencing of Robo4 in microvascular ECs may be correlated with the upregulation of GJs. Furthermore, a radiation-induced downregulation of the connexin 43 gene and protein expression was observed after treatment of ECs with 10 Gy of γ-radiation, indicating that IR may regulate the expression of GJs at the gene level. However, we cannot completely rule out that high-dose IR may directly disrupt the EC junction proteins, probably causing the decreased expression of the connexin 43 protein. To find out if the upregulation of connexins after Robo4 knockdown could offer any protection against high-dose irradiation, on the contrary, we noticed a significant downregulation of the mRNA expression of the connexins (Fig. 2H) compared to the shControl group after IR. This finding was similar to connexin 43 protein expression (Fig. 2H and I) following Robo4 silencing and irradiation treatments, indicating that Robo4 plays a crucial role in regulating GJs. We designed a cytofluorimetric assay to determine the potential role of Robo4 knockdown and irradiation in modulating GJIC. Donor cells were treated with calcein and PKH26, and recipient cells were treated with or without gamma radiation before co-culture. Both Robo4 depletion and irradiation caused a significant increase in the transfer of Calcein Blue AM among the injured cells (Fig. 2J).

### EC Robo4 silencing increases the degradation of TJ molecules after exposure to a high dosage of IR

**Fig. 3:**
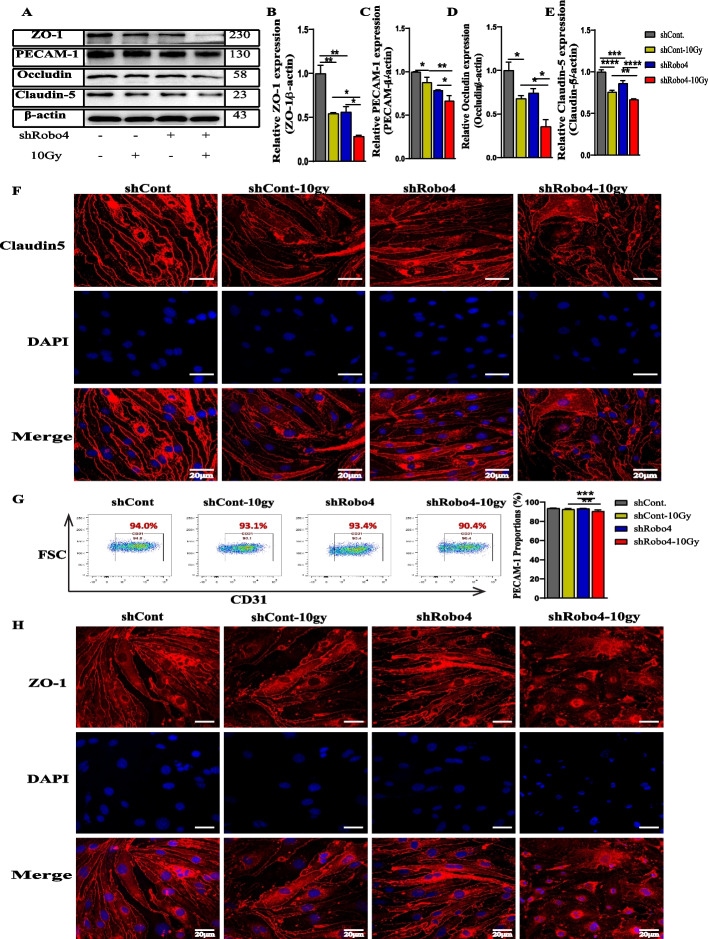
Robo4 silencing degrades the expression and organization of TJ molecules after γ-radiation exposure. **A–E** Western blotting analysis of claudin-5, occludin, PECAM-1, and ZO-1 normalized with endogenous beta-actin protein. The relative protein expression were quantified using Image J software. **F** Immunostaining for claudin-5 (red) and DAPI staining (blue) in irradiated Robo4 knockdown and control microvascular EC samples. **G** Flow cytometric analysis of PECAM-1 expression levels after Robo4 knockdown and irradiation. **H** Confocal imaging of ZO-1 (red) and DAPI (blue) in Robo4 silenced and controlled ECs with or without gamma radiation exposure. Scale bars = 20 µm. For all experiments, *n* ≥ 3, and error bars represent std. *P < 0.05, **P < 0.01, ***P < 0.001, ****P < 0.0001

TJs play a critical physiological role in the permeability of the vasculature by regulating the tightness of the barrier [15]; hence, we hypothesize that IR exposure may induce further degradation of TJs in Robo4-depleted microvascular ECs. Using western blot analysis, protein samples collected from ECs after Robo4 knockdown and irradiation were examined to determine the effect of IR in Robo4-silenced ECs. The protein expression of claudin-5, occludin, PECAM-1, and ZO-1 was significantly decreased in both shControl and shRobo4 groups after IR. However, these reductions of TJs were more pronounced in shRobo4 ECs, confirming the downregulation or destruction of TJs in shRobo4 ECs compared to the control groups after IR (Fig. 3A–E). We conducted immunofluorescence staining to determine the expression and distribution of claudin-5 and ZO-1 in shRobo4 and shControl ECs following irradiation. We observed a reduction in claudin-5 and ZO-1 levels and distributions of Robo4 knockdown cells relative to the control groups. Even though IR tends to weaken the expression of claudin-5 and ZO-1, it became worse in cells expressing less Robo4 (Fig. 3F, H). Consistently, flow cytometric analysis of CD31 expression revealed a significant decrease after Robo4 knockdown and irradiation (Fig. 3G). These findings suggest that Robo4 may play a crucial role in regulating TJs, especially after IR-induced disruption of the microvascular EC junctions.

### Enhanced expression of Robo4 mitigates IR-induced depletion of VE-cadherin, improves the integrity of microvascular EC junctions, and decreases permeability

**Fig. 4:**
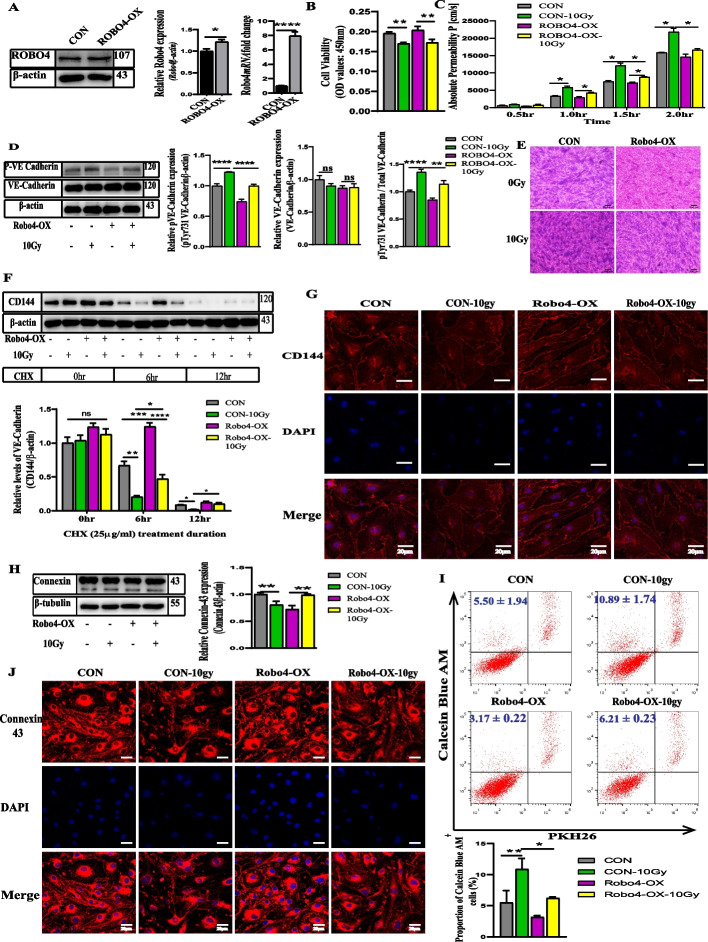
Overexpression of Robo4 improves IR-induced downregulation of VE-cadherin and decreases the permeability of the EC monolayer. **A** Confirmation of Robo4 overexpression after establishing a stable cell line using western blot and qPCR analysis, respectively. **B** Effect of Robo4 overexpression and irradiation on cell survival and proliferation of ECs. **C** and **E** Functional analysis of Robo4 overexpression by FITC-dextran (70 kDa) permeability assay and EC monolayer crystal violet staining after irradiation. **D** Western blot analysis of total and pY-731 VE-cadherin after Robo4 overexpression and irradiation; the relative protein expression of phosphorylated Y-731 and total VE-cadherin normalized with endogenous beta-actin protein; The ratio of pY-731 VE-cadherin to total VE-cadherin. Cycloheximide treatment and immunoblotting were conducted as in (**F**). The CD144 band intensity was normalized to actin and then normalized to the *t* = 0 controls. **G** Confocal microscopic images of VE-cadherin (red) and DAPI (blue). **H** Western blot analysis showing connexin 43 expression levels in Robo4 overexpression microvascular ECs after gamma radiation treatment. **J** Confocal images show immunofluorescence of connexin 43 (red) in Robo4 overexpression microvascular ECs, with or without irradiation. Nuclei appear in blue. **I** Flow cytometric analysis to determine EC GJ coupling ability in vitro. For all experiments, scale bars = 20 µm, *n* ≥ 3, and error bars represent std. *P < 0.05, **P < 0.01, ***P < 0.001, ****P < 0.0001

As we illustrated above, Robo4 silencing makes ECs susceptible to the damaging effects of IR; it is only ideal for investigating if overexpression of Robo4 could perhaps protect the vascular integrity after IR. So, we used lentiviral-mediated technology to enhance the expression of Robo4 in our microvascular ECs. The endogenous expression of Robo4 mRNA and protein levels in these cells, depicted in (Fig. 4A), increased significantly after successful LV-Robo4 (45395-1) transduction. While 10 Gy gamma radiation affects cell viability and proliferation, Robo4 overexpression does not affect the microvascular EC viability (Fig. 2B). Using FITC-Dextran permeability and endothelial monolayer staining assays, we measured the extent of permeability after exposing Robo4 overexpression ECs and the control cells to 10 Gy gamma radiation. However, the level of permeability significantly decreased in the Robo4 OE group after IR compared to the irradiated control group (Fig. 4E). Since VE-Cadherin is the critical regulator of vascular permeability, we wanted to assess whether the enhanced Robo4 expression could inhibit its irradiation effects. To this end, we irradiated both Robo4 OE and the control groups with 10 Gy, collected the protein lysates 24 h post-irradiation, and the expression levels of total and Y731 phosphorylated VE-cadherin were analyzed using western blot analysis. Our findings showed no significant changes in total VE-cadherin expression levels between the non-irradiated and irradiated Robo4 OE groups, like the control groups. We observed an increased relative amount of Y731 phosphorylated VE-cadherin after exposure to IR. However, Robo4 overexpression led to a further decrease in phosphorylated VE-cadherin levels compared to the control after irradiation (Fig. 4D). From cycloheximide chase assay, we also determined the turnover of VE-cadherin following Robo4 overexpression and irradiation, after 3 h, cells were treated with cycloheximide and incubated for 0, 6, and 12 h. The half-life of VE-cadherin protein was higher in Robo4 overexpression ECs than in their controls (Fig. 4F). Furthermore, from immunocytochemical analysis, Robo4 overexpression causes the upregulation of VE-cadherin expression after irradiation. It improves the destabilization effect of high-dose IR on microvascular ECs (Fig. 4G). These findings suggest that Robo4 overexpression may play a crucial role in regulating vascular barrier permeability through the AJs after radiation injury.

### Robo4 overexpression enhances connexin 43 stability in response to radiation-induced injury

A decrease in the Robo4 level increased the expression of a critical GJ molecule, connexin 43, that plays an essential role in vascular permeability [35]. To investigate the effect of Robo4 overexpression on GJs, we used immunoblotting to determine whether Robo4 could affect the regulation of connexin 43 in microvascular ECs. As expected, the expression level of connexin 43 decreased significantly after Robo4 overexpression. However, after irradiation, connexin 43 levels were upregulated in ECs overexpressing Robo4 compared with the control (Fig. 4H). Consistent with our western blot findings, immunofluorescence staining showed an improved connexin 43 distribution and organization after Robo4 overexpression and irradiation than the irradiated control (Fig. 4J). Enhanced expression of Robo4 reduced the GJIC properties of ECs exposed to radiation (Fig. 4I). Based on these outcomes, we can say that Robo4 supports GJs when there is IR-induced stress.

### Overexpression of Robo4 mitigates irradiation-induced degradation of TJs and stabilizes claudin-5 and ZO-1 distribution

**Fig. 5:**
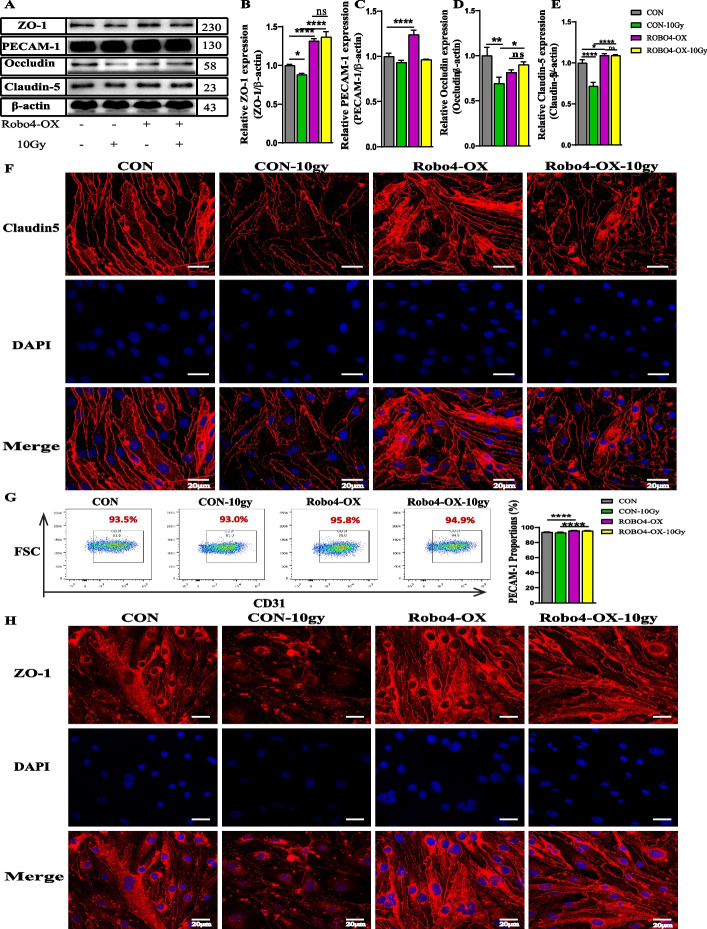
Enhanced expression of Robo4 ameliorates the downregulation of TJs induced by IR. **A**–**E** Western blotting analysis showing the protein expression levels of claudin-5, occludin, PECAM-1, and ZO-1 in Robo4 overexpressed bEnd3 cells after irradiation. We used β-actin as a loading control. **F** and **H** Localization of claudin-5 and ZO-1 in microvascular EC samples by confocal microscopy following IR, respectively. The nuclear stain is DAPI. **G** Assessment of CD31 expression in the microvascular ECs 24 h post-irradiation using flow cytometry. Scale bars = 20 µm. For all experiments, *n* ≥ 3, and error bars represent std. *P < 0.05, **P < 0.01, ***P < 0.001, ****P < 0.0001

After demonstrating that Robo4 knockdown increases the degradation of TJ molecules after exposure to IR, we hypothesized that Robo4 overexpression in microvascular ECs could ameliorate the injury caused by IR. To determine the impact of lentiviral-mediated enhanced expression of Robo4 on TJs, we employed western blotting to analyze the effect of Robo4 overexpression on ZO-1, PECAM-1, occludin, and claudin-5 levels without IR. In addition to occludin, these cells’ relative expression of ZO-1, PECAM-1, and claudin-5 increased after Robo4 overexpression. After exposure to 10 Gy of gamma radiation, we re-evaluated the protein expression using western blotting analysis on these tight junctional molecules. Our results showed that Robo4 overexpression potentiates significantly increased endothelial ZO-1, occludin, and claudin-5 after IR treatment compared to the control groups (Fig. 5A–E).

Furthermore, we performed immunofluorescence staining to ascertain the expression and distribution of ZO-1 and claudin-5 in Robo4-enhanced ECs with or without irradiation. We noticed an apparent alteration in the distribution and localization of ZO-1 and claudin-5 in ECs with Robo4 overexpression and the control. Also, we found that exposure to IR has a less degradative effect on the localization and expression of ZO-1 and claudin-5 in ECs with Robo4 overexpression than the control (Fig. 5F, H). Similarly, flow cytometry analysis revealed a higher CD31 expression in Robo4 overexpression ECs than the control upon irradiation (Fig. 5G). These findings suggest that overexpression of Robo4 may improve the permeability of endothelial monolayer by modulating the expression and localization of these TJs after high-dose IR treatment.

## Discussion

Bone marrow transplantation usually requires radiation preconditioning to ablate the recipient’s hematopoietic cells. Still, this irradiation regime also adversely affects the bone marrow microenvironment constituents that tightly control the fate of hematopoietic stem and progenitor cells [36, 37]. Vascular and sinusoidal ECs are key components of the bone marrow microenvironment that are essential in sustaining the homeostasis of the bone marrow and promoting the hematopoietic reconstruction of the HSC niche [38, 39]. A high dose of irradiation treatment is known to destroy the structural and functional integrity of the vascular network in the bone marrow, where sinusoidal and arterial HSCs reside [40]. Only a handful of research exists on the underlying mechanism of irradiation-induced injury in the endothelium, and the recovery mechanism remains unclear. Bone marrow microvascular endothelium acts as a gateway for hematopoietic cells to enter or exit the bone marrow to enrich the circulating blood with new blood cells, hence the need to investigate the mechanisms that modulate the gateway [41]. Endothelial junctional molecules regulate the permeability of ECs. The endothelial junctions include AJs, TJs, and GJs [4, 5, 14]. The AJs and TJs comprise endothelial monolayers’ basic lateral sealing components. GJs contain intercellular channels that allow chemical and electrical communication between adjacent cells [22–24]. Endothelial TJs are composed of occludins, claudin family proteins, and other junctional adhesion molecules linked to the actin cytoskeleton by the zonula occludens (ZO) proteins [7, 11, 15]. AJs predominantly comprise VE-cadherins, linked to the actin filaments through interactions with catenins [16–18]. To explore the effect of irradiation preconditioning on vascular integrity, we established an in vitro microvascular endothelial injury model to determine the impact of a radiation-induced injury on the endothelial monolayer. We discovered that a high dose (10 Gy) of γ-radiation exposure had a detrimental effect on EC junctions. This study found that the mRNA and protein levels of key junctional molecules declined after irradiation, except for VE-cadherin, with no considerable difference in total expression levels. Moreover, the localization of claudin-5, connexin 43, and VE-cadherin was disrupted, suggesting a direct effect of IR on the EC junctions. The idea that IR increases endothelial permeability is not entirely new. A recent report revealed that irradiation-induced endothelial monolayer permeability is not dependent on VEGF signaling or activity of proteasomes and lysosomes but rather through ADAM10-mediated degradation of VE-cadherin [13]. To ascertain the mechanism involved in regulating endothelial hyperpermeability after irradiation, we detected the mRNA and protein expression of Robo4 after irradiation and noticed a significant upregulation. Here, we hypothesized that Robo4 could have a protective effect on microvascular ECs following a high dose of IR exposure. Recent studies have shown that Robo4, an endothelial-specific marker, maintains vascular stability and integrity by inhibiting VEGF-induced angiogenesis and permeability under pathological conditions [25]. Also, others have reported that Robo4 interacts with UNC5B and maintains blood vessel integrity by counteracting VEGF signaling in ECs [42]. However, to the best of our knowledge, there has been no study about Robo4 regulating irradiation-induced injury to the endothelium. We have shown that Robo4 knockdown exacerbates the endothelial permeability induced by irradiation. Studies have shown that VE-cadherin is a major modulator of microvascular endothelial permeability in the bone marrow [43]. Antibody-mediated inhibition of VE-cadherin led to increased permeability in both arterioles and sinusoids of the bone marrow under normal conditions and upon low-dose irradiation [44, 45]. Silencing Robo4 in microvascular ECs, we observed a substantial decrease in the expression of total VE-cadherin following irradiation, suggesting a potential role of Robo4 in regulating AJs. However, we know that vascular permeability-inducing agents stimulate their effects through the phosphorylation of VE-cadherin and its concomitant internalization [46]. It has been shown that activated protein kinase Cα (PKCα) can induce the phosphorylation of VE-cadherin at Tyr731 resulting in AJs disorientation and loss of connective function in ECs [47, 48]. In this study, we also found that Robo4 depletion enhances the phosphorylation of Y-731 VE-cadherin, leading to the degradation and delocalization of VE-cadherin at the EC junctions upon irradiation. These results are consistent with previous studies demonstrating that Robo4 in microvascular ECs may inhibit the destruction of VE-cadherin during endotoxemia [27, 30]. Considering VE-cadherin disruption and increased permeability in Robo4-knockdown ECs following irradiation, we speculate that Robo4 overexpression could alleviate the damaging effects of IR on the endothelial barrier. Previous studies showed that the overexpression of Robo4 decreased endothelial permeability induced by LPS or TNFα [30, 49]. Our results confirm the initial hypothesis that overexpression of Robo4 decreases endothelial permeability upon irradiation. We observed that Robo4 overexpression did not significantly alter VE-cadherin expression levels but reduced the extent of Y731 VE-cadherin phosphorylation and mitigated VE-cadherin destabilization at the EC junctions after irradiation.

No previous study has articulated the role that EC TJs play in the bone marrow microenvironment, but several reports have indicated that these TJs modulate paracellular permeability across the blood–brain barrier [50, 51]. The loss of ZO-1, the first identified protein of TJ, induces its disorganization. Claudin-5 and occludin are directly involved in the endothelial barrier and wall function [52, 53]. Moreover, PECAM-1, a transmembrane protein, engages in homotypic interaction with cognate molecules on neighboring ECs to modulate endothelial TJs integrity [54]. Our findings demonstrated that the expression levels of claudin-5, occludin, PECAM-1, and ZO-1 were significantly suppressed in Robo4 shRNA-transfected microvascular ECs. Similar to our results, Cai et al. reported that Robo4 depletion could increase blood-tumor barrier permeability by downregulating the expression and distribution of claudin-5, occludin, and ZO-1 [49]. We also found that exposure of Robo4 knockdown ECs to IR decreases the expression of TJs proteins. Even though there were no considerable changes in claudin-5 and ZO-1 distribution after Robo4 knockdown, we observed irregular patterning and disrupted organization of these TJs following irradiation. Several reports suggest that IR disrupts claudin-5, occludin, PECAM-1, and ZO-1 [55, 56], but what is unknown is the role Robo4 plays in regulating these TJ molecules against the damaging effects of irradiation. Apart from occludin, the expression of claudin-5, PECAM-1, and ZO-1 were upregulated after lentiviral-mediated Robo4 overexpression. One previous study also reported the upregulation of these molecules after Robo4 overexpression in hCMEC/D3 cells [49]. In other reports, VE-cadherin can control TJs by increasing the transcription of claudin-5 and occludin genes [57, 58]. Therefore, it is possible that with reduced VE-cadherin levels, TJs cannot function properly or vice versa, even if occludin and claudin-5 levels are elevated through other mechanisms. Considering the interaction between AJs and TJs, we speculated that overexpression of Robo4 may mitigate the degradation of TJs through VE-cadherin after exposure to γ-radiation. Our data showed that upregulation of Robo4 enhanced ZO-1 and claudin-5 orientation and localization and the expression of TJs upon irradiation. Thus, our findings support a novel Robo4-mediated regulatory mechanism of irradiation-induced permeability of the endothelium.

Transmembrane connexin proteins regulate vascular leakage by forming intercellular gap junctional and hemichannel paracrine communication pathways [59, 60]. Although the existence of GJs in bone marrow has been known for more than four decades [34, 61], their function in regulating bone marrow hematopoiesis remains an open question. Other studies suggest that bone marrow stromal cells form a dynamic syncytium via connexin GJs to regulate the homeostasis of HSCs [62, 63]. Connexin 43 is the major connexin expressed by ECs and maintains the normal function of the vasculature [64]. In the present study, we demonstrated the correlation between Robo4 and GJs in ECs for the first time.

Interestingly, the knockdown of microvascular Robo4 increased connexin 43 expression and localization at the junctions compared to the normal ECs with high cytoplasmic localization of connexin 43, synthesized in the endoplasmic reticulum [65, 66]. Several past studies indicated that a decrease in connexin 43 levels paralleled the decline in microvascular permeability and vice versa. They attributed their findings to the inverse correlation between the expression of VE-cadherin and connexin 43 [35, 67]. Thus, the present results show for the first time that a reduction in Robo4 levels correlates with a concomitant increase in GJs expression and microvascular permeability. Previous reports have shown that low-dose (0.5 and 5 Gy) IR induces acute and persistent upregulation of the connexin 43 gene and protein expression in microvascular ECs by increasing gap junctional communication and causing hemichannel opening [68–70].

In contrast, our results demonstrated a significant decrease in endothelial connexin 43 expression after receiving 10 Gy of gamma radiation, even in Robo4 knockdown ECs. This contradiction can result from differences in IR doses, suggesting that a high dosage of IR can directly degrade the transmembrane connexins in endothelial monolayers. Connexin in ECs exhibit rapid turnover with half-lives ranging from one to five hours, and given the organization of GJs, even a modest change in the expression of connexin levels at the plasma membrane can result in a quick alteration in connexon hemichannel formation and cell–cell communication. Accordingly, connexins can respond readily to several conditions due to the plasticity of their expression and the transient dynamics of the formed hemichannels and GJs [71, 72]. Thus, the trafficking and turnover of connexin must be tightly regulated in response to different cellular stresses and signals, explaining the disparity between connexin 43 expression patterns and GJIC findings. In addition, we further analyzed the impact of Robo4 overexpression on GJs, and our results showed the downregulation of connexin 43. We identified that Cx43 is downregulated in tandem with a decrease in the permeability of enhanced Robo4 microvascular ECs. Thus, reduced Cx43 expression contributes to increases and reductions in VE-cadherin and vascular permeability, respectively. However, our data suggest that Robo4 overexpression reduces the direct degradation of connexin 43 at the EC junctions caused by irradiation but instead stimulates its expression levels. The data allude to the possibility that endothelial GJs play a significant role in restoring endothelial barrier integrity during recovery from irradiation injury.

## Conclusion

Our study demonstrates that Robo4 ameliorates microvascular permeability induced by a high dose of γ-radiation, and Robo4 knockdown worsens the depletion of EC junctions after irradiation. Our data also identified Robo4 as a crucial molecule that repairs the bone marrow vascular and sinusoidal niche after preconditioning treatment.

## Data Availability

All data generated or analyzed during this study are included in this published article.

## Abbreviations

CD: Cluster of differentiation
EDTA: Ethylenediaminetetraacetic acid
HSCT: Hematopoietic stem cell transplantation
LPS: Lipopolysaccharide
PECAM-1: Platelet endothelial cell adhesion molecule-1
PGF: Poor graft function
Robo4: Roundabout protein 4
TBST: Tris-buffered saline with 0.1% Tween
TBI: Total body irradiation
VEGF: Vascular endothelial growth factor
